# Machine learning-based construction of immunogenic cell death-related score for improving prognosis and response to immunotherapy in melanoma

**DOI:** 10.18632/aging.204636

**Published:** 2023-04-06

**Authors:** Guoyin Li, Huina Zhang, Jin Zhao, Qiongwen Liu, Jinke Jiao, Mingsheng Yang, Changjing Wu

**Affiliations:** 1 College of Life Science and Agronomy, Zhoukou Normal University, Zhoukou, Henan, China; 2 Key Laboratory of Modern Teaching Technology, Ministry of Education, Shaanxi Normal University, Xi’an, Shaanxi, China; 3 Academy of Medical Science, Zhengzhou University, Zhengzhou, Henan, China

**Keywords:** melanoma, immunogenic cell death, machine learning, prognostic model, immunotherapy

## Abstract

Background: Immunogenic cell death (ICD) is a form of regulated cell death (RCD) which could drive the activation of the innate and adaptive immune responses. In this work, we aimed to develop an ICD-related signature to facilitate the assessment of prognosis and immunotherapy response for melanoma patients.

Methods: A set of machine learning methods, including consensus clustering, non-negative matrix factorization (NMF) method and least absolute shrinkage and selection operator (LASSO) logistic regression model, and bioinformatics analytic tools were integrated to construct an ICD-related risk score (ICDscore). CIBERSORT and ESTIMATE algorithm were used to evaluate the infiltration of immune cells. The 'pRRophetic' package in R and 6 cohorts of melanoma patients receiving immunotherapy were used for therapy sensitivity analyses. The predictive performance between ICDscore with other mRNA signatures were also compared.

Results: The ICDscore could predict prognosis and immunotherapy response in multiple cohorts, and displayed superior performance than other forms of cell death-related signatures or 52 published signatures. The melanoma patients with low ICDscore were marked with high infiltration of immune cells, high expression of immune checkpoint inhibitor-related genes, and increased tumor mutation burden.

Conclusions: In conclusion, we constructed a stable and robust ICD-related signature for evaluating the prognosis and benefits of immunotherapy, and it could serve as a promising tool to guide decision-making and surveillance for individual melanoma patients.

## INTRODUCTION

An ideal anti-cancer therapy is still an ongoing pursuit for most researchers and clinicians. The extensive research efforts in past decades have resulted in the emergence of immune-checkpoint inhibitors (ICIs) and various types of targeted therapy. However, not all patients, even with the same tumor type and at the same stage, responded well to these anti-tumor agents. For instance, only 4% uveal melanoma patients with metastasis showed partial response to nivolumab plus ipilimumab, as reported in a recent published real-life, retrospective study [1]. In a multicenter, randomized phase 3 study, only 36-37% and 13% of melanoma patients at advanced stage showed an objective response to pembrolizumab and ipilimumab, respectively [2]. Given the complexity of the human genome and the heterogeneity of the tumor itself, personalized therapy might be an alternative option to deal with the aforementioned problem. Some efforts have been made to classify patients into subgroups with a different risk score, based on the generation of gene expression signatures [3]. The most successful signature might be the 21-gene expression assay, which helps to identify a subgroup of hormone-receptor–positive, HER2-negative, axillary node–negative breast cancer patients with a high risk of recurrence and to guide adjuvant chemotherapy for these patients [4, 5].

Inducing the death of tumor cells is the ultimate purpose of cancer treatment. Multiple forms of regulated cell death (RCD) have been identified in cancer, including apoptosis, autophagy, ferroptosis, necroptosis, and proptosis [6]. Immunogenic cell death (ICD) is one kind of RCD but unique in its capability to trigger antigen-specific adaptive immunological responses [7]. Various types of anti-cancer therapy, including chemotherapy and radiotherapy, could drive the occurrence of ICD [7]. In addition, induction of ICD also becomes a strategy in the design of anti-cancer agents in various types of tumors including melanoma. For example, Li et al. generated polymer micelles (named PP_IR780-ZMS_) containing IR780 dye and manganese zinc sulfide nanoparticles (ZMS), and revealed that PP_IR780-ZMS_ could maximize ICD in melanoma and augment the infiltration of cytotoxic T cells (CD8+) and helper T cells (CD4+) [8]. Zhang et al. synthesized a nano-inducer (named DOX/ADS NP) via the self-assembly of doxorubicin (DOX) and dermatan sulphate derivative (ADS) grafted by aromatic thioketal (ATK) [9]. This nano-inducer could enhance ICD and activate the immune response in melanoma, leading to shrinkage of tumor [9].

Although some previous studies have attempted to develop signatures, based on some form of RCD, like autophagy and ferroptosis [10–13], and classify melanoma patients into subgroups with a different risk score, little is known about the application of ICD-based signature in melanoma. Since melanoma is supposed to be an immunogenic tumor whose proliferation and progression have a tight relationship with immune cells [14, 15], and ICIs have been shown by clinical trials to improve the survival benefits of melanoma patients and have been approved for clinical use globally [16–18], we hypothesize that a novel ICD-based risk score might be a better signature in predicting prognosis and immunotherapy efficiency of melanoma patients.

In this work, unsupervised clustering of melanoma patients, based on 34 ICD-related genes summarized by a previous study [19], indicated that these patients could be classified into two subgroups with distinct overall survival (OS). Subsequently, we constructed an ICD-related risk score (ICDscore) and found that melanoma patients with high ICDscore had apparently shorter OS and poor sensitivity to immunotherapies. More importantly, we compared ICDscore with other signatures developed on other forms of RCD and noticed that ICDscore had better performance in predicting OS and immunotherapy efficiency. We also compared ICDscore with 52 previously published signatures, and the results indicated that ICDscore had certain advantages over these signatures regarding prognosis predictability.

## MATERIALS AND METHODS

### Public data acquisition and processing

The TCGA-SKCM cohort was downloaded from the Cancer Genome Atlas (TCGA) database (https://portal.gdc.cancer.gov). The GSE35640, GSE54467, GSE22153, and GSE65904 were obtained from the Gene Expression Omnibus (GEO) database (https://ncbi.nlm.nih.gov/gds). The gene expression data and clinical information of the Peking University Cancer Hospital (PUCH) study, Riaz17 study, Liu19 study, VanAllen15 study and Gide19 study were downloaded from the GitHub website (https://github.com/), as reported in Cui’s study [20]. All datasets were processed as described in our previous work [21]. All datasets used in this work were downloaded from public databases, an extra ethical approval was not necessary.

### Construction of ICDscore

**Figure 1:**
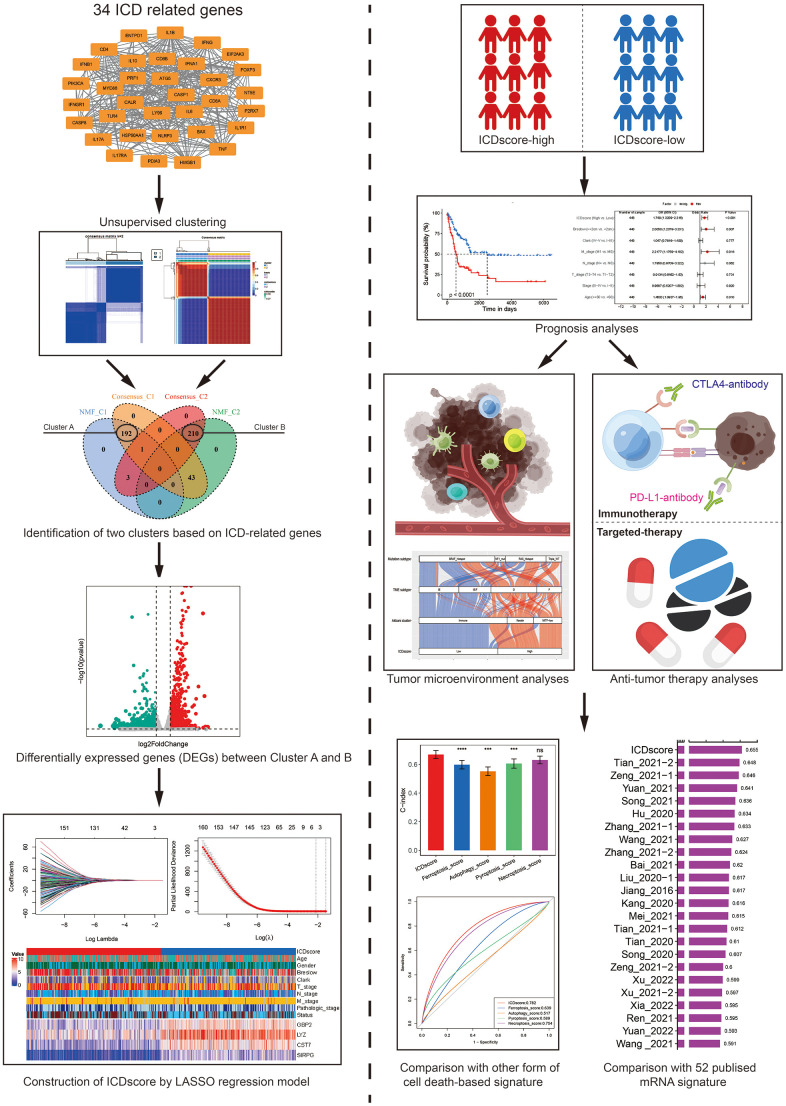
**The work flow for the construction of ICDscore.** Two clustering methods (consensus clustering and NMF clustering) were used for the molecular subtyping of melanoma patients, based on the gene expression of 34 ICD related genes. Two clusters (named as Cluster A and B) were identified and DEGs between these two clusters were analyzed. The LASSO regression model, multivariate Cox analyses were then used for the construction of ICDscore. The association between the ICDscore and prognosis, tumor immune microenvironment, or immunotherapy response was comprehensively investigated. The performance between ICDscore and other signatures was compared.

The flow to generate the ICDscore is summarized in Figure 1. Specifically, melanoma patients were firstly stratified into two clusters (namely cluster A and B) via the consensus clustering and non-negative matrix factorization (NMF) clustering methods, based on the gene expression of 34 ICD-related genes. For the consensus clustering, the ‘ConcensusClusterPlus’ package in R was used [22], and the parameters used in the analyses were maxK = 10, reps = 1, 000, pItem = 0.8, pFeature = 1, clusterAlg = “pam”, corUse = “complete.obs”, seed = 123456. For the NMF clustering, the “NMF” package in R was used [23], and the ranks were set from 2 to 10 to do the NMF rank survey. The differentially expressed genes (DEGs) between cluster A and B were assessed by the “limma” package in R. Univariate Cox regression analyses were then performed to identify DEGs which had a significant p-value < 0.1 in the TCGA-SKCM and GSE65904 cohorts. Subsequently, a total of 182 DEGs were then input into a Least absolute shrinkage and selection operator (LASSO) regression model in GSE65904. 4 key genes were generated, and their corresponding coefficients were obtained via multi-variate Cox analysis. The risk score for each patient was calculated by the following formula: score = - 0.17244 * GBP2 - 0.14202 * LYZ - 0.24610 * CST7 - 0.19525 * SIRPG. The ICDscore of patients in each cohort was calculated with the formula: ICDscore = (score-Min) / absolute (Max), as reported in our previous studies [21, 24].

### Immune profile analysis

The infiltration ratio of 22 immune cells in patients was calculated by the CIBERSORT algorithm in R software, as reported in our previous study [21]. The immunescore and stromalscore of each patient were calculated by the ‘estimate’ package in R [25].

### Enrichment analysis

Gene Set Enrichment Analysis (GSEA) of SKCM patients were performed by the ‘clusterProfiler’ package in R. The *c5.go.bp.v2022.1.Hs.symbols.gmt* was chosen as the gene set database. The ‘GseaVis’ package in R was used for visualization [26].

### Statistical analysis

All the data were processed, analyzed and visualized by R software (version 4.1.3). In addition to the packages mentioned above, other packages in R used in this work included “tidyverse”, “survival”, “msigdbr”, “dplyr”, “org.Hs.eg.db”, “ggplot2”, “glmnet”, “scales”, “aplot ”, “survivalROC”, “ggrepel”, “enrichplot”, “corrplot”, “survminer”, “timeROC”, “rms”, “pec”, “ggalluvial”, “VennDiagram”, “ggh4x”, “patchwork”, “pRRophetic”, and “CompareC”. The Kaplan-Meier method was used for prognosis analyses. The Correlation analyses were conducted with the Pearson method. The comparison of categorical variables between two groups was conducted with the chi-square test. The continuous variables were compared with the Wilcoxon rank-sum test. A value of p < 0.05 was considered to be statistically significant (*, p < 0.05; **, p < 0.01; ***, p < 0.001; ****, p < 0.0001).

## RESULTS

### Unsupervised clustering of ICD-related genes in melanoma

**Figure 2:**
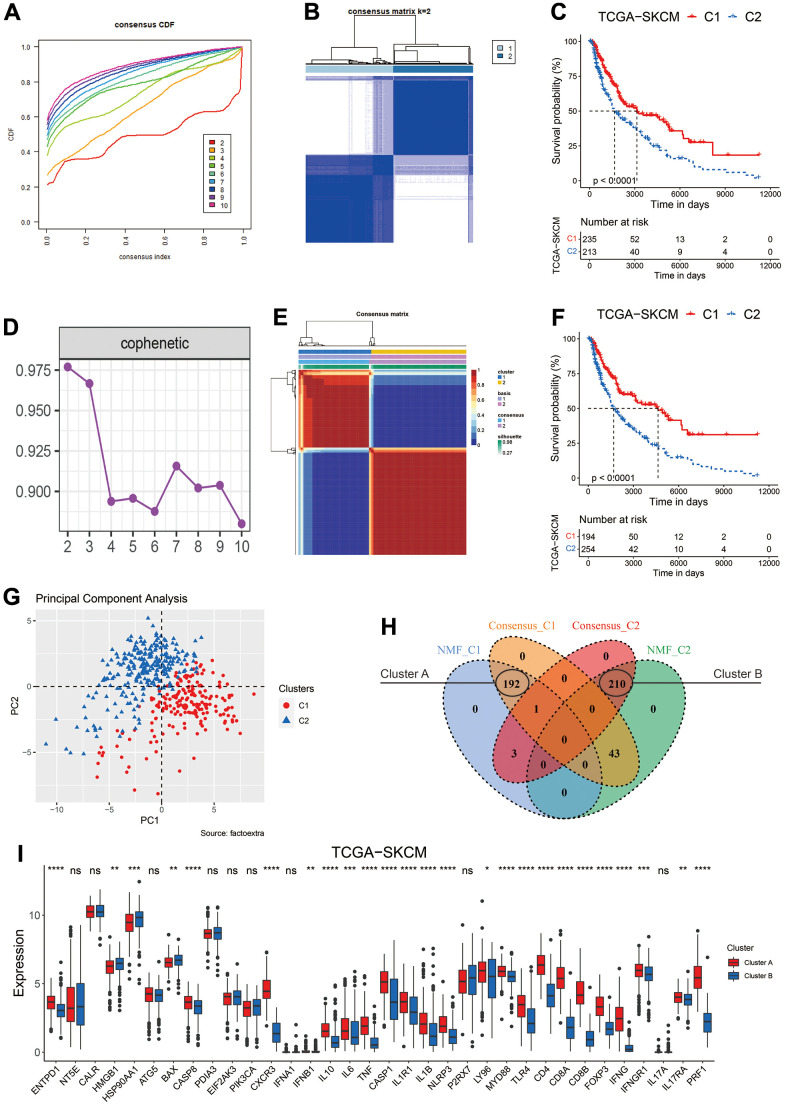
**Clustering of melanoma patients based on ICD related genes.** (**A**) The cumulative distribution function (CDF) curve of consensus clustering for k = 2 to 10. (**B**) Heatmap depicts consensus clustering solution (k = 2) for 34 ICD related genes in melanoma patients from the TCGA-SKCM dataset. (**C**) Kaplan–Meier curves of OS in the C1 and C2 subtypes (the consensus clustering method) of melanoma patients. (**D**) The optimal rank was selected as 2 since the cophenetic coefficient firstly started decreasing at this point. (**E**) Heatmap of NMF clustering results of melanoma patients from the TCGA-SKCM dataset. (**F**) Kaplan–Meier curves of OS in the C1 and C2 subtypes (the NMF method) of melanoma patients. (**G**) Principal component analysis (PCA) on the expression level of 34 ICD related genes in clusters classified by NMF method. (**H**) Venn diagram to identify melanoma patients in the C1 cluster (Cluster A) and C2 cluster (Cluster B) defined by both clustering methods. (**I**) Gene expression comparison of 34 ICD related genes between Cluster A and B in the TCGA-SKCM cohort. Ns, not significant; *p < 0.05; **, p < 0.01; ***, p < 0.001; ****, p < 0.0001.

Based on the expression of 34 ICD-related genes identified by Abhishek D. Garg et al. [19], two unsupervised clustering methods were employed to stratify melanoma patients in the TCGA-SKCM cohort. For the Consensus Clustering method, k = 2 was selected as the optimal parameter for further analyses, based on the consensus matrix for k = 2 to 10 and the consensus cumulative distribution function (CDF) plot (Figure 2A, 2B and Supplementary Figure 1A–1H). Two hundred thirty-five melanoma patients were classified into C1 cluster and showed significantly prolonged median overall survival (OS) than those in the C2 cluster (Figure 1C). For the Nonnegative Matrix Factorization (NMF) method, rank = 2 was chosen as the most suitable number of subgroups according to the cophenetic coefficient and the consensus matrix for different rank numbers (Figure 2D, 2E and Supplementary Figure 1I, 1J). 194 patients were stratified into C1 cluster and exhibited better prognosis (Figure 2F, p < 0.0001). No matter which method was applied, an obviously different distribution between was observed the C1 and C2 clusters via the PCA plots (Figure 2G and Supplementary Figure 1K). As shown in Figure 2H, 192 melanoma patients were stratified in the C1 cluster by both methods and named Cluster A. Meanwhile, 210 patients were grouped in the C2 cluster by both methods and were named as Cluster B. The median OS of melanoma patients in Cluster A reached 4634 days, and was considerably longer than that of patients in Cluster B (1766 days, Supplementary Figure 1L, p < 0.0001). In addition, the transcriptional expression of most of the ICD-related genes was significantly elevated in patients of Cluster A (Figure 2H).

### The biological features of melanoma patients in the two clusters

**Figure 3:**
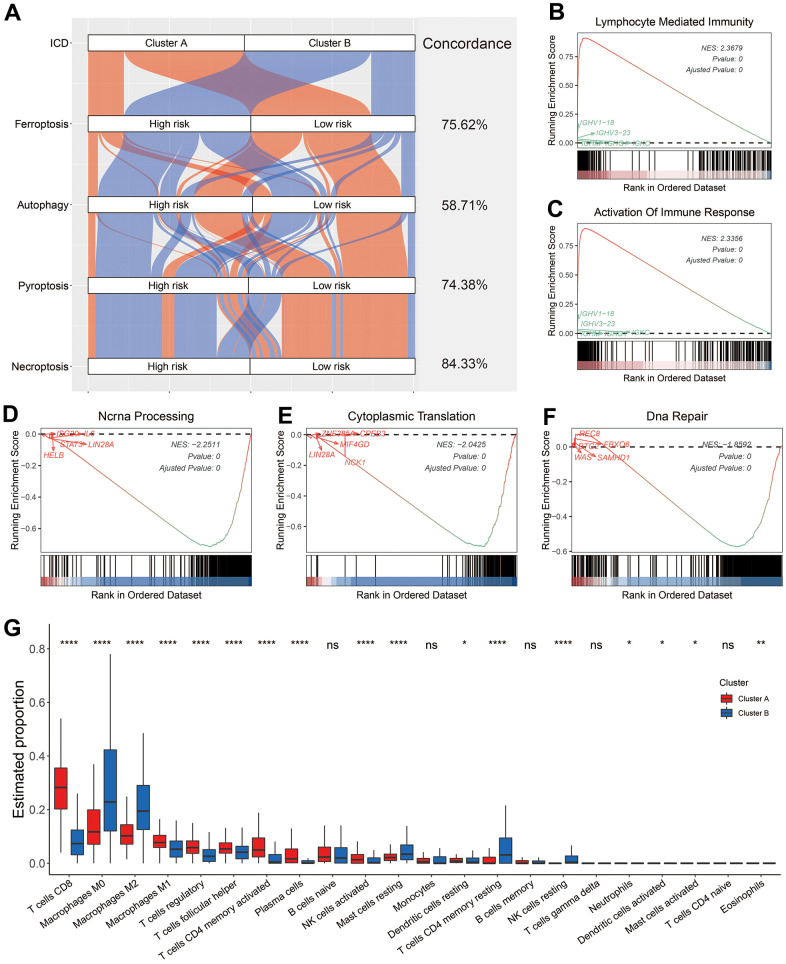
**Biological features of melanoma patients in Cluster A and B.** (**A**) Sankey diagram showed the connection degree between ICD-based classification and other forms of cell death-based classification. (**B**–**F**) Examples of GSEA results of melanoma patients in Cluster A (**B**, **C**) and B (**D**–**F**). (**G**) Distribution of 22 types of infiltrating immune cells in melanoma patients in the Cluster A and B. Ns, not significant; *p < 0.05; **, p < 0.01; ***, p < 0.001; ****, p < 0.0001.

Besides ICD, various other forms of cell death have been identified, such as autophagy, ferroptosis and necroptosis [27]. These forms of cell death have some shared regulators or signaling components, but also have unique biological characteristics. Some previous studies tried to classify melanoma patients into high- and low-risk subgroups based on the features of these forms of cell death [10–13]. As shown in Figure 3A, most the melanoma patients in Cluster A were labeled as low risk when they were classified based on the ferroptosis-, autophagy-, proptosis-, or necroptosis-related signatures. The concordance between ICD-based classification and other forms of cell death-based classification was 75.62% (ferroptosis), 58.71% (autophagy), 74.38% (proptosis), and necroptosis (84.33%). To characterize the biological features of patients in Cluster A and Cluster B, GSEA was employed. The results showed that melanoma patients in Cluster A showed enrichment in immune related pathways (Supplementary Table 1) like lymphocyte mediated immunity (Figure 3B) and activation of immune response (Figure 3C). On the other hand, patients in the Cluster B showed an enrichment in the processing and translation of RNA (Supplementary Table 1 and Figure 3D, 3E), and in DNA repair (Supplementary Table 1 and Figure 3F). Consistently, melanoma patients had an obviously higher level of infiltration of anti-tumor immune components, such as CD8 T cells, M1 macrophage, and activated memory CD4 T cells (Figure 3G), whereas some pro-tumor immune components, like M2 macrophage, resting mast cells, and resting memory CD4 T cells, showed a significantly higher infiltration in patients from the Cluster B (Figure 3G).

### Construction of ICD-related signature in melanoma

**Figure 4:**
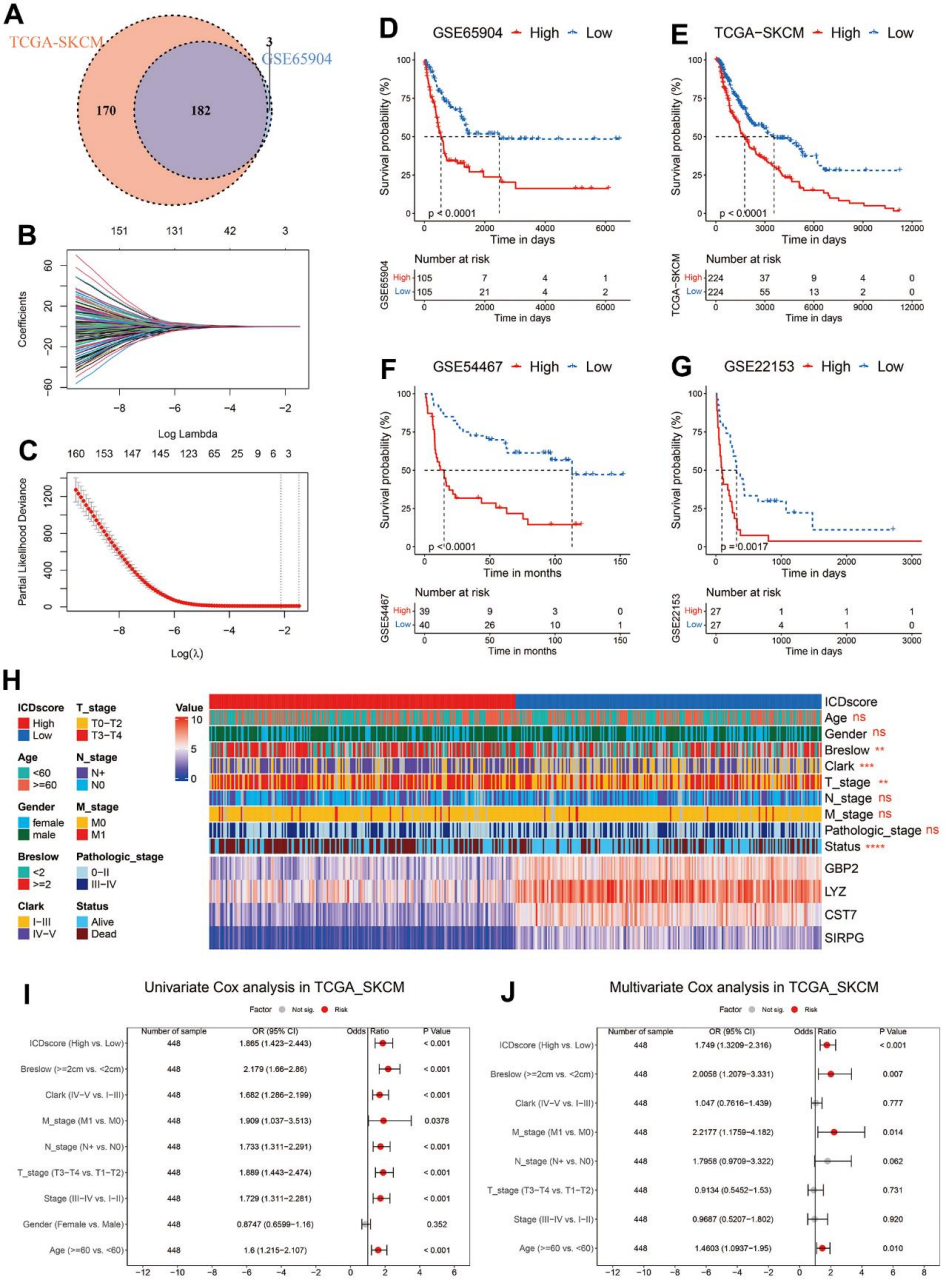
**Construction of ICDscore.** (**A**) Venn diagram to screen DEGs with a significant prognostic p-value < 0.1 in the TCGA-SKCM and GSE65904 cohorts. (**B**, **C**) The LASSO Cox regression model was constructed from 182 DEGs, and the tuning parameter (λ) was calculated based on the partial likelihood deviance with ten-fold cross validation. 4 signature genes were identified according to the best fit profile. (**D**–**G**) Kaplan–Meier curves of OS in melanoma patients from ICDscore-high and ICDscore-low subgroups of GSE65904 (**D**), TCGA-SKCM (**E**), GSE54467 (**F**), and GSE22153 (**G**) datasets. (**H**) Clinical characteristics and RNA expression level of 4 crucial genes in melanoma patients from ICDscore-high and ICDscore-low subgroups of the TCGA-SKCM dataset. (**I**) Univariate analysis of ICDscore and other clinical characteristics in TCGA-SKCM dataset. (**J**) Multivariate analysis shows ICDscore, breslow depth, M stage and age were independent prognostic factors. Ns, not significant; *p < 0.05; **, p < 0.01; ***, p < 0.001; ****, p < 0.0001.

Unsupervised clustering revealed that ICD-related genes could help to stratify melanoma patients into subgroups with distinct prognoses and tumor microenvironment (TME) (Figures 2, 3). To construct a signature that help to recognize these two subgroups, we first analyzed the differentially expressed genes (DEGs) between the patients in Cluster A and those in Cluster B. A total of 1, 038 DEGs were identified in the TCGA-SKCM cohort with the criteria of absolute logFC (fold change) ≥ 1 and adjusted p-value < 0.05 (Supplementary Table 2). To better reflect the difference between these two clusters, only DEGs (genes marked with red, Supplementary Table 2) with absolute logFC ≥ 2 were selected for further analyses. The subsequent univariate Cox analyses in TCGA-SKCM and GSE65904 indicated that 182 DEGs had a significant p-value < 0.1 in both datasets (Figure 4A). The 182 DEGs were then input into a LASSO regression model in GSE65904 as described in the Method section (Figure 4B, 4C). Four crucial genes were generated, and they were guanylate binding protein 2 (GBP2), lysozyme (LYZ), cystatin F (CST7), and signal regulatory protein gamma (SIRPG). The ICDscore was calculated based on the transcriptional expression and coefficient of these 4 genes, as demonstrated in the Method section. When melanoma patients were divided into two groups based on the median value of ICDscore in each cohort, those patients in the high-ICDscore subgroup showed a significantly shorter OS in the training (GSE65904, Figure 4D) and validating (TCGA-SKCM, GSE54467, and GSE22153, Figure 4E–4G) datasets. The forest plot of meta-analysis (Supplementary Figure 2A) also indicated ICDscore as a vital risk factor for melanoma patients. Besides, melanoma patients with high ICDscore also exhibited considerably shorter progression-free survival (PFS, Supplementary Figure 2B, 2C). As shown in Figure 4H, patients with low ICDscore had apparently higher expression of all the four genes. In addition, the high-ICDscore subgroup had a significantly higher percentage of melanoma patients with deeper Breslow depth (p < 0.01), advanced Clark level (p < 0.001), advanced T stage (p < 0.01), and dead status (p < 0.0001). Correspondingly, melanoma patients with deeper Breslow depth (Supplementary Figure 2G), advanced Clark level (Supplementary Figure 2H), advanced T stage (Supplementary Figure 2I) exhibited a significantly higher level of ICDscore, and no difference of ICDscore level was observed in melanoma patients divided by gender (Supplementary Figure 2E), age (Supplementary Figure 2F), N stage (Supplementary Figure 2J), M stage (Supplementary Figure 2K) or clinical stage (Supplementary Figure 2L). Besides, univariate and subsequent multivariate Cox analyses revealed that ICDscore could serve as an independent prognostic factor (Figure 4I, 4J).

### Immune landscape of ICDscore-stratified melanoma patients

**Figure 5:**
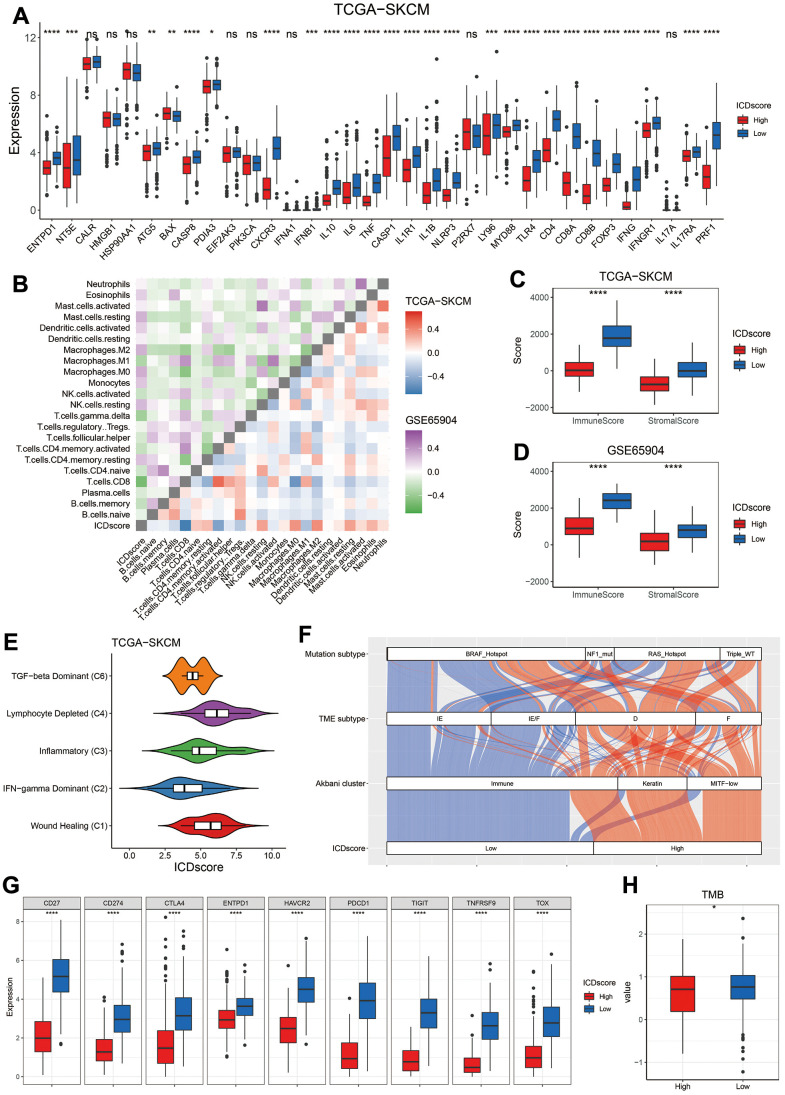
**Immune profile of ICDscore-based classification.** (**A**) Gene expression comparison of 34 ICD related genes between ICDscore-high and ICDscore-low subgroups in the TCGA-SKCM cohort. (**B**) Correlation analyses between ICDscore and infiltration level of 22 immune cells in the TCGA-SKCM and GSE65904 datasets. (**C**, **D**) Comparison of immunescore (**C**) and stromalscore (**D**) between ICDscore-high and ICDscore-low subgroups in the TCGA-SKCM and GSE65904 datasets. (**E**) Box plot showing a difference in the value of ICDscore across the five subtypes for melanoma patients in the TCGA_SKCM dataset. (**F**) Sankey diagram showed the connection degree between ICDscore-based classification and Akbani cluster, TME subtype and mutation subtype in the TCGA-SKCM dataset. (**G**) Box plot showing a difference in the expression of multiple exhausted T cell markers or immune-checkpoint markers between ICDscore-high and ICDscore-low subgroups in the TCGA-SKCM cohort. (**H**) Box plot showing a difference in the TMB between ICDscore-high and ICDscore-low subgroups in the TCGA-SKCM cohort. Ns, not significant; *p < 0.05; **, p < 0.01; ***, p < 0.001; ****, p < 0.0001.

As shown in Supplementary Figure 2C, 94.79% of patients in the Cluster A were classified into low-ICDscore subgroup, while 90.52% of patients in the Cluster B were into the high-ICDscore subgroup. Most of ICD-related genes showed a significantly elevated expression in patients from the low-ICDscore group (Figure 5A). Correlation analyses in both the TCGA-SKCM and GSE65904 cohorts revealed that ICDscore had a strong negative correlation with CD8 T cells (R = -0.65 in TCGA-SKCM cohort and -0.53 in GSE65904 cohort, Figure 5B), and significantly positive correlation with NK cells resting, M0 macrophage, M2 macrophage, or mast cell resting (Figure 5B). In addition, melanoma patients from the ICDscore-low subgroup had an apparently higher immunescore and stromalscore (Figure 5C, 5D), suggesting a higher infiltrative level of immune and stromal cells.

The relationship between ICDscore-based classification and other method-based classification was also investigated. TCGA research network divided cancer patients into six clusters [28]. As shown in Figure 5E, the C2 cluster (INF-gamma dominant) had the lowest ICDscore, whereas the C4 (lymphocyte depleted) and C1 (wound healing) clusters had relatively high ICDscore. Besides, most melanoma patients in the low-ICDscore subgroup were categorized into the immune cluster, which was defined on the basis of consensus hierarchical clustering of 1500 genes [29]. Similarly, 83.26% of patients in the low-ICDscore subgroup were labeled as ‘Immune-Enriched, Fibrotic’ (IE/F) or ‘Immune-Enriched, Non-Fibrotic’ (IE), based on the characterization of TME by 29 functional gene expression signatures (Fges) [30]. In addition, melanoma patients were also divided into four subtypes based on some mutated genes, and they were *B-Raf Proto-Oncogene, Serine/Threonine Kinase (BRAF)* hotspot, *RAS* hotspot, *Neurofibromin 1* (*NF1)* mutant, and Triple-WT (wild-type) [29]. The distribution of ICDscore-high or ICDscore-low patients in BRAF hotspot, NF1 mutant, or RAS hotspot subtype was similar (Figure 5F, Chi-square test, p > 0.05), but a significantly higher ratio of ICDscore-high patients was Triple-WT (Figure 5F, Chi-square test, p = 0.0465).

Meanwhile, the expression of multiple exhausted T cell markers or immune-checkpoint markers, including CD27, CD274 (also referred as PDL1), cytotoxic T-lymphocyte associated protein 4 (CTLA4), programmed cell death 1 (PDCD1), hepatitis A virus cellular receptor 2 (HAVCR2, also named as TIM3), T cell immunoreceptor with Ig and ITIM domains (TIGIT), thymocyte selection associated high mobility group box (TOX), TNF receptor superfamily member 9 (TNFRSF9), and ectonucleoside triphosphate diphosphohydrolase 1 (ENTPD1) [31, 32], were dramatically elevated in patients with low ICDscore (Figure 5G), suggesting these patients might be able to benefit from immune checkpoint inhibitors (ICIs). Further, tumor mutation burden (TMB), another biomarker in predicting immunotherapy efficiency [33], was also significantly higher in the ICDscore-low subtype (Figure 5H, p < 0.05).

### Evaluation of anti-tumor therapy in ICDscore-based subgroups

**Figure 6:**
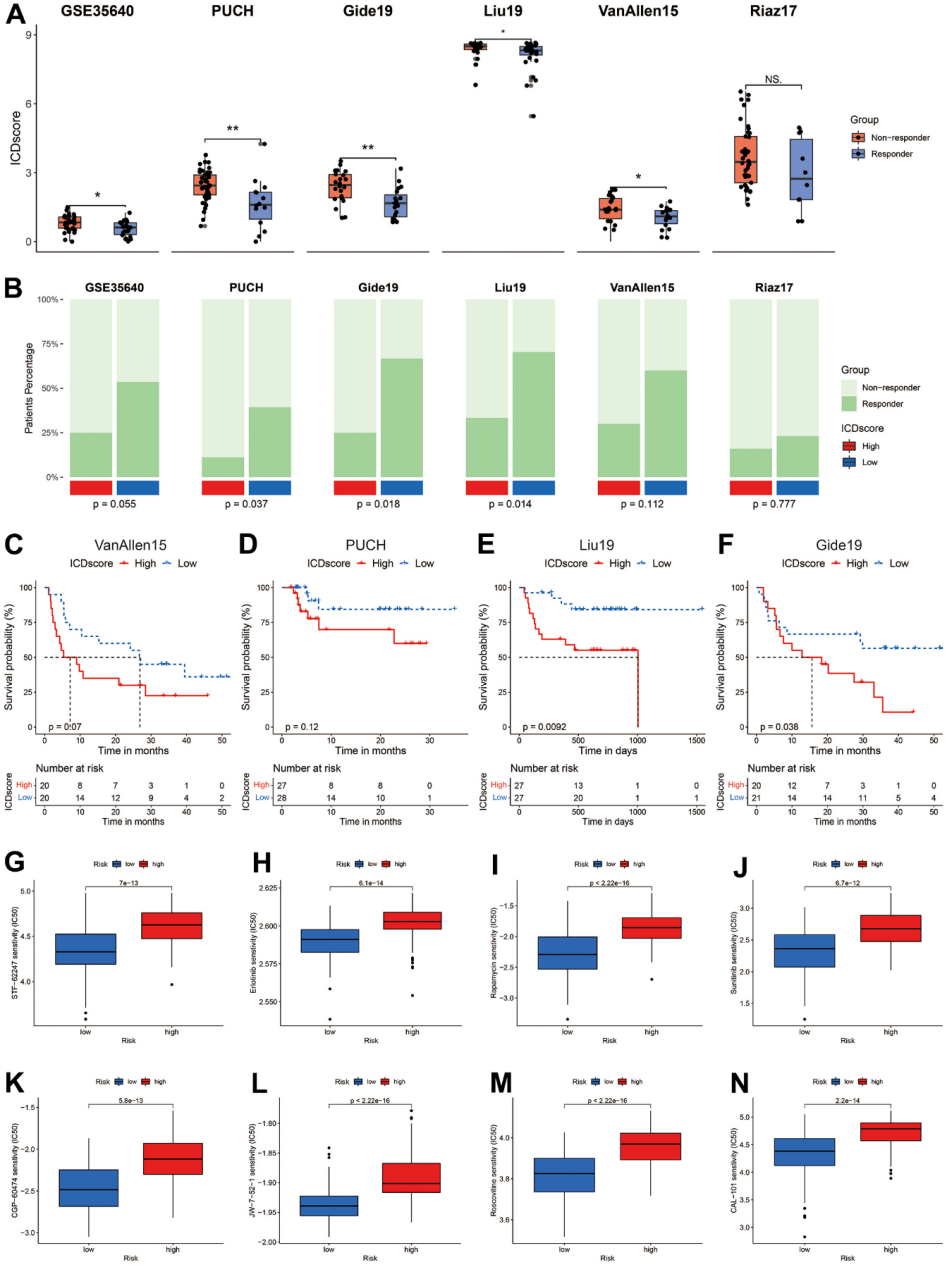
**Sensitivity evaluation to anti-cancer therapy.** (**A**) ICDscore of melanoma patients receiving immunotherapy in GSE35640, PUCH, Gide19, Liu19, VanAllen16, and Riaz17 cohorts. (**B**) Ratio of patients responding or not responding to immunotherapy in ICDscore-high and ICDscore-low subgroups of GSE35640, PUCH, Gide19, Liu19, VanAllen16, and Riaz17 cohorts. (**C**–**F**) Kaplan–Meier curves of OS in melanoma patients from ICDscore-high and ICDscore-low subgroups of VanAllen15 (**C**), PUCH (**D**), Liu19 (**E**), and Gide19 (**G**) cohorts. (**G**–**N**) Box plot showing a difference in the IC50 values of STF-62247 (**G**), Erlotinib (**H**), Rapamycin (**I**), Sunitinib (**J**), CGP-60474 (**K**), JW-7-52-1 (**L**), Roscovitine (**M**), CAL-101 (**N**) between ICDscore-high and ICDscore-low melanoma patients. Ns, not significant; *p < 0.05; **, p < 0.01.

To support the hypothesis that ICDscore might help to predict immunotherapy efficiency, several cohorts of melanoma patients receiving various forms of immunotherapy were investigated. As shown in Figure 6A, melanoma patients who responded to adoptive T-cell therapy (ACT) treatment (GSE35640), anti-PD-1 (PUCH, Gide19, and Liu19 cohorts) or anti-CTLA-4 (VanAllen15 cohort) showed a significant lower ICDscore than those who were non-responders to immunotherapies. In the Riaz17 cohort, melanoma patients who responded to nivolumab (anti-PD-1 agent) had lower ICDscore than the non-responders, but the difference did not reach significance (Figure 6A). When melanoma patients were divided into ICDscore-high and ICDscore-low subgroups by the median value of ICDscore in each cohort, the patients in the ICDscore-low subgroup showed a higher object responsive rate (ORR) to immunotherapies, and the difference reached significance in the PUCH (p = 0.037), Gide19 (p = 0.018), and Liu19 (p = 0.014) cohorts (Figure 6B). In four cohorts with survival information available, patients in the ICDscore-low subgroup showed apparently longer OS than those in the ICDscore-high subgroup (Figure 6C–6F).

We further manipulated the ‘pRRophetic’ package in R software to estimate the drug sensitivity of melanoma patients in ICDscore-classified subgroups. As shown in Supplementary Table 3, ICDscore had a strong correlation (absolute R > 0.5, p-value < 0.05) with patients’ sensitivity to 34 anti-cancer agents. The top 8 agents, according to the coefficiency, were JW-7-52-1, Roscovitine (CDK inhibitor), Rapamycin (mTOR inhibitor), CGP-60474 (CDK inhibitor), Erlotinib (EGFR inhibitor), CAL-101 (PI3K inhibitor), STF-62247 (autophagy inducer), Sunitinib (Multi-kinase inhibitor), and melanoma patients with high ICDscore had a dramatically higher IC50 value than those with low ICDscore (Figure 6G–6N).

### Comparison of ICDscore and other gene expression-based prognostic signatures

**Figure 7:**
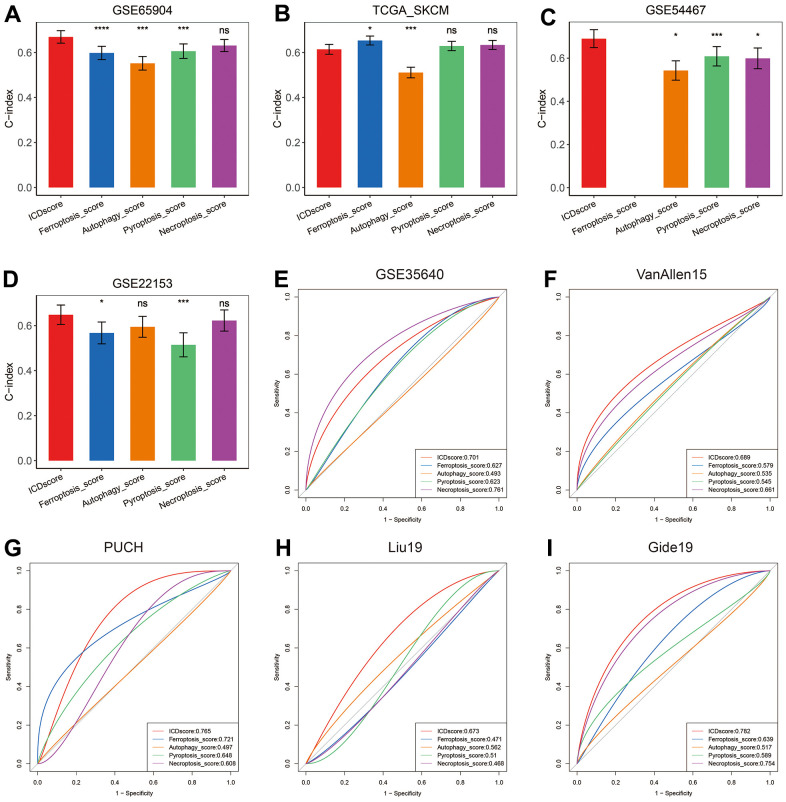
**Comparison between ICDscore and other forms of cell death-based signatures.** (**A**–**D**) The performance of ICDscore was compared with other forms of cell death-based signatures in predicting prognosis in the GSE65904 (**A**), TCGA-SKCM (**B**), GSE54467 (**C**), and GSE22153 (**D**) datasets. Statistic tests: two-sided z-score test. Data were presented as mean ± 95% confidence interval [CI]. (**E**–**I**) The performance of ICDscore was compared with other forms of cell death-based signatures in predicting immunotherapy efficiency in GSE35640 (**E**), VanAllen15 (**F**), PUCH (**G**), Liu19 (**H**), and Gide19 (**I**) cohorts. Ns, not significant; *p < 0.05; **, p < 0.01; ***, p < 0.001; ****, p < 0.0001.

As shown in Figure 3A, unsupervised clustering of melanoma patients based on ICD-related genes had 58.71% to 84.33% concordance with other forms of cell death-based classification. The C-index [95% confidence interval] of ICDscore was 0.669 [0.641 – 0.697], 0.614 [0.592 – 0.636], 0.690 [0.649 – 0.732], and 0.648 [0.605 – 0.692] in GSE65904, TCGA-SKCM, GSE54467 and GSE22153 cohort, respectively. When compared with clinical features of melanoma patients, the C-index of ICDscore was lower than that of Breslow depth, T stage, and Clark level, but higher than that of the rest clinical features (Supplementary Figure 2M). However, no significance was observed in the comparison of ICDscore and clinical features of melanoma patients (Supplementary Figure 2M). As displayed in Figure 7A–7D, ICDscore showed certain improved accuracy than ferroptosis-, autophagy-, proptosis-, or necroptosis-based signature (except for comparison between ICDscore and ferroptosis-related risk score in TCGA-SKCM, Figure 7B). Besides, the AUC of ICDscore in predicting efficiency to immunotherapies was 0.701, 0.689, 0.765, 0.673, and 0.782 in GSE35640, VanAllen15, PUCH, Liu19, and Gide19 cohorts, respectively (Figure 7E–7I), and was the highest in these cohorts except for GSE35640, suggesting a stable and robust performance in various independent datasets.

**Figure 8:**
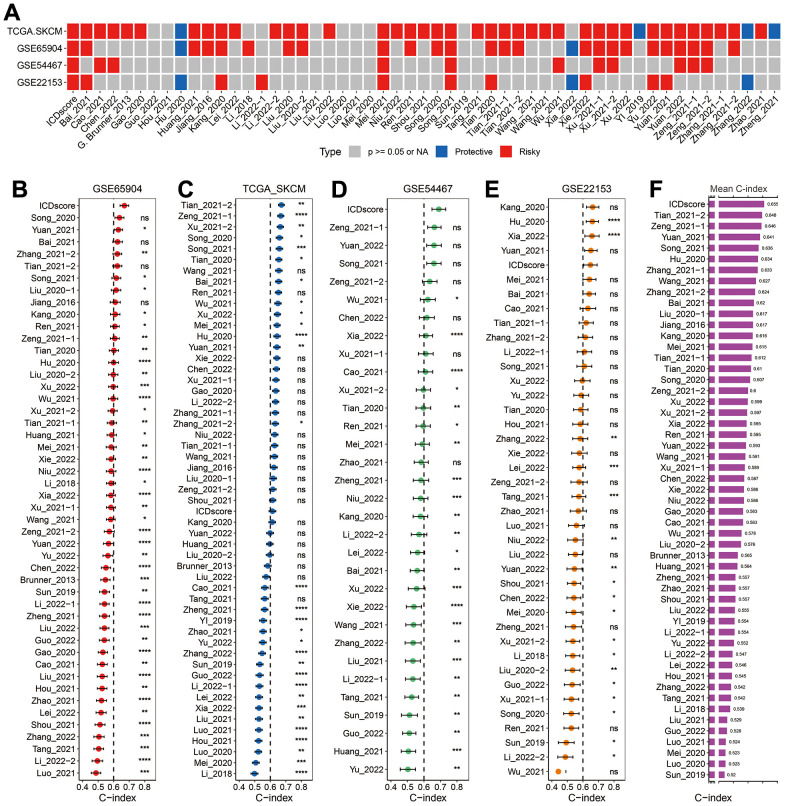
**Comparison between ICDscore and other published signatures.** (**A**) Univariate Cox regression analysis of ICDscore and 52 published signatures in GSE65904, TCGA-SKCM, GSE54467, and GSE22153 datasets. (**B**–**E**) C-index analyses of ICDscore and 52 published signatures in GSE65904 (**B**), TCGA-SKCM (**C**), GSE54467 (**D**), and GSE22153 (**E**) datasets. Statistic tests: two-sided z-score test. Data are presented as mean ± 95% confidence interval [CI]. (**F**) The average C-index of ICDscore and 52 published signatures across all the 4 datasets. Ns, not significant; *p < 0.05; **, p < 0.01; ***, p < 0.001; ****, p < 0.0001.

In addition to cell death-based signatures, many other gene expression signatures, for example, hypoxia- or CD8 T cell-related signatures, had been constructed to predict the prognosis of melanoma patients [24, 34]. By searching the PubMed website with the following query method “((signature[Title]) OR (classifier[Title])) AND (melanoma[Title])”, 52 mRNA signatures were ultimately enrolled for further comparison (Supplementary Table 4). These signatures were associated with DNA methylation, RNA methylation, oxidative stress, immune cell infiltration, metastasis, ferroptosis, proptosis or other biological process. Univariate Cox analyses revealed that only ICDscore, tumor immune-relevant (TIR) signature (Mei_2021) [35] and immune-associated genes (IAGs) signature (Song_2021) [36] had significant prognostic relevance across the four datasets (Figure 8A). The C-index of ICDscore displayed the best performance in GSE65904 and GSE54467 (Figure 8B, 8D), and ranked the fifth in the GSE22153 cohort (Figure 8E). In the TCGA-SKCM cohort, the C-index of ICDscore was not the highest, but was greater than 0.6 (Figure 8C). Besides, ICDscore had the highest average C-index (0.655) in all the four datasets (Figure 8F).

## DISCUSSION

The capability of ICD to drive anti-cancer immune response provokes researchers’ interest in developing ICD-related risk model that predicts prognosis and response to immunotherapy in tumors [37, 38]. In both these two studies, ICD-associated subtyping systems were helpful for the development of precision immunotherapy. According to several published clinical studies, the ORR of previously untreated patients with metastatic melanoma is from about 10% to 57.6%, suggesting most of melanoma patients are insensitive to the ICIs [16, 18]. Given the tight association between ICD and immune response, ICD-related risk score might be helpful to identify melanoma patients potentially benefiting from immunotherapy.

In this work, two unsupervised clustering methods both showed that melanoma patients could be classified into two subgroups based on the expression of 34 ICD-related genes (Figure 2B–2E). To minimize the potential bias of methodology, the clustering results of two methods were intersected, and melanoma patients grouped into C1 cluster by both methods were renamed as ‘Cluster A’, whereas those grouped into C2 cluster by both methods were renamed as ‘Cluster B’ (Figure 2H). The patients in the Cluster A had significantly elevated expression of most innate or adaptive immune system-related genes (Figure 2I). Consistently, patients in the Cluster A had a significant enrichment in most immune-related pathways like activation of immune response (Figure 3C) and was marked by dramatically higher infiltration of CD8 T cells (Figure 3G). Once ICD was triggered by anti-cancer therapy, the release of many damage-associated molecular patterns (DAMPs), such as cell surface-exposed calreticulin (CALR), extracellular adenosine triphosphate (ATP), could drive the activation and maturation of innate and subsequent adaptive immune cells [39]. Thus, it would be reasonable to propose that a higher expression of innate or adaptive immune system-related genes and infiltration of CD8 T cells might facilitate the occurrence of ICD and cause the amplification of anti-tumor effects induced by immune cells like cytotoxic T cells [37, 40, 41].

Further, the DEGs between Cluster A and B were input into a LASSO regression model, and 4 crucial genes were generated for the construction of ICDscore (Figure 4A–4C). All the 4 genes participate in immune activities. GBP2 is a member of the p65 guanine-binding protein (GBP) family, which includes interferon-induced large GTPase that exhibits antiviral activity through the innate immune response [42, 43]. Lysozymes, encoded by LYZ, has antibacterial activity and constitutes the first line of defense [44, 45]. CST7 is predominantly expressed in immune cells and tends to attenuate the granule-dependent cytotoxicity of natural killer (NK) cells and T cells [46–48]. SIRPG is expressed by T cells and interacts with CD47 on the surface of the cell, and modulates immune responses [49, 50]. Prognostic analyses in the testing dataset and three independent validating datasets revealed that high ICDscore correlated with significantly shorter OS and PFS (Figure 4D–4G and Supplementary Figure 2B, 2C). In addition, ICDscore could serve as an independent prognostic factor (Figure 4I, 4J and Supplementary Table 5). Notably, ICDscore also had superior performance than other cell death related signatures in predicting prognosis of melanoma patients (Figure 7A–7D). Moreover, we retrieved another 52 published mRNA signatures correlating with various biological activities. Univariate Cox regression displayed that only ICDscore and two other signatures maintained prognostic significance across all the four cohorts, suggesting most of the signatures have not been thoroughly validated (Figure 8A). It should be pointed out that many models had good performance in the training dataset but relatively poor performance in external validating datasets (Xu_2021-2 or Song_2020, for example) [51, 52]. Compared with other signatures, ICDscore exhibited stable performance across multiple cohorts and its average C-index was the highest, suggesting a better capability in predicting prognosis of melanoma patients (Figure 8B).

The value of ICDscore in guiding anti-cancer therapy was also investigated. On one hand, melanoma patients with low ICDscore were marked with significantly high infiltration of immune cells, particularly CD8 T cells (Figure 5B, 5D), suggesting an “immune-hot” phenotype (Figure 5E, 5F) [53]. These patients also exhibited high expression of ICI-related genes, such as PD-L1 (CD274) and CTLA4 (Figure 5G), whose high expression is generally associated with response to immunotherapy [54]. In addition, patients in the ICDscore-low subgroup also had higher TMB (Figure 5H), which could increase the production of mutation-derived neoantigens and contribute to activation of cytotoxic T cells [55]. In 6 independent cohorts in which melanoma patients receiving immunotherapy, patients who responded to immunotherapy displayed lower ICDscore (Figure 6A). Similarly, patients in the low-ICDscore subgroup generally had higher ORR to immunotherapy (Figure 6B) and longer OS (Figure 6C–6F). The AUC of ICDscore in predicting response to immunotherapy was from 0.673 to 0.782, and was higher than that of other cell death related signatures (Figure 7E–7I), suggesting a better performance than these signatures. On the other hand, ICDscore exhibited strong positive correlation with multiple targeted drugs like rapamycin and sunitinib (Figure 6G–6N and Supplementary Table 3). Although some preclinical models or clinical trials had been conducted to evaluate the benefit of some of these drugs in melanoma [56–58], the results are far from satisfactory in clinical practice. The ICDscore might be helpful in selecting suitable patients when conducting clinical trials.

At last, the limitation of this work should be pointed out that the ICDscore was developed and validated in a retrospective method; thus, evidence from a well-designed perspective analysis is required before the model be applied in clinics.

## CONCLUSIONS

In conclusion, we constructed a stable and powerful ICD-related signature for evaluating the prognosis and benefits of immunotherapy, based on the integration of a set of bioinformatics tools. The ICDscore showed certain superiority than other mRNA signatures and served as a promising tool to guide decision-making and surveillance for individual melanoma patients.

## Supplementary Material

Supplementary Figures

Supplementary Table 1

Supplementary Table 2

Supplementary Table 3

Supplementary Table 4

Supplementary Table 5
