# Supplementary material for: Machine learning-based construction of immunogenic cell death-related score for improving prognosis and response to immunotherapy in melanoma
Source: Aging (Albany NY). 2023 Apr 6;15(7):2667–88. doi: 10.18632/aging.204636 (PMC10120887; doi:10.18632/aging.204636)
Supplement: Supplementary Figures [file aging-15-204636-s001.pdf]

SUPPLEMENTARY FIGURES

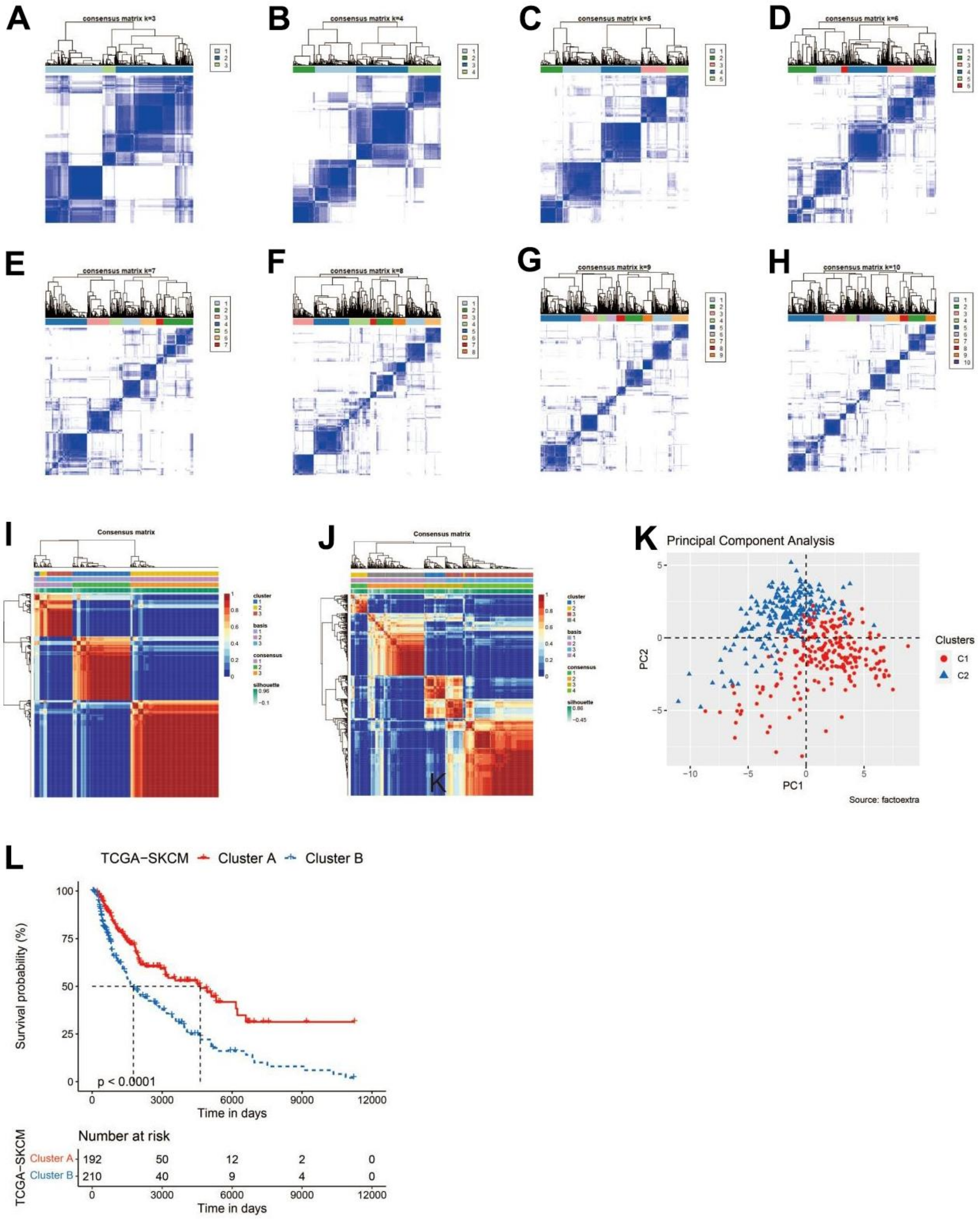

Supplementary Figure 1. (**A**–**H**) Heatmap depicts consensus clustering solution for 34 ICD related genes in melanoma patients from the TCGA-SKCM dataset, when the k = 3 (**A**), 4 (**B**), 5 (**C**), 6 (**D**), 7 (**E**), 8 (**F**), 9 (**G**), and 10 (**H**). (**I**, **J**) Heatmap of NMF clustering results of melanoma patients from the TCGA-SKCM dataset, when the k = 3 (**I**) and 4 (**J**). (**K**) Principal component analysis (PCA) on the expression level of 34 ICD related genes in clusters classified by consensus clustering method. (**L**) Kaplan–Meier curves of OS in the Cluster A and B of melanoma patients.

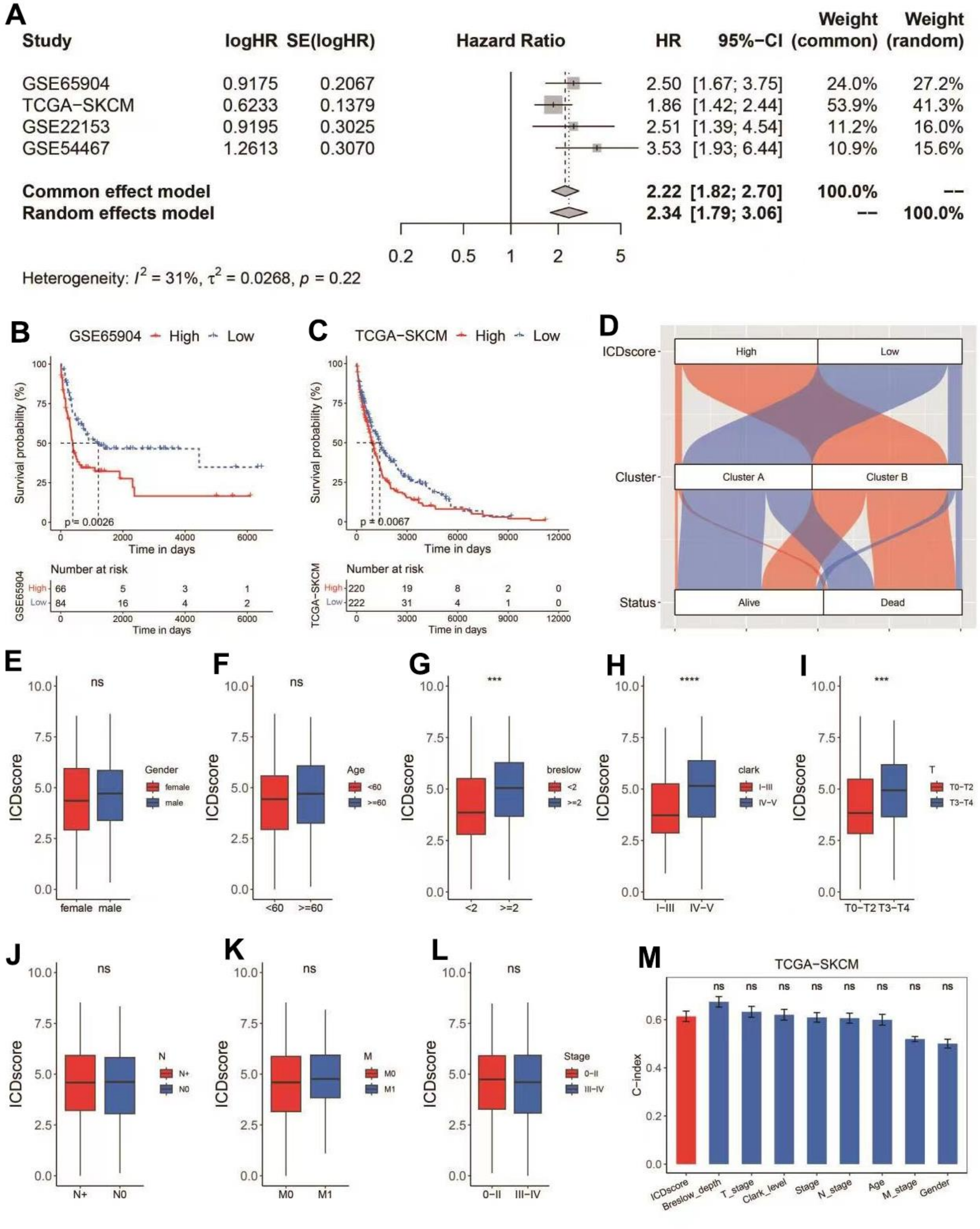

Supplementary Figure 2. (**A**) Forest plot for the outcome of ICDscore in meta-analysis of melanoma patients using common or random model. (**B**, **C**) Kaplan–Meier curves of PFS in melanoma patients from ICDscore-high and ICDscore-low subgroups of GSE65904 (**A**) and TCGA-SKCM (**B**). (**C**) Sankey diagram showed the connection degree between ICD-based classification and Cluster A/B. (**D**) Sankey diagram showed the connection degree between ICDscore and patient clustering and survival status. (**E**–**L**) The distribution of ICDscore in melanoma patients divided by gender (**E**), age (**F**), breslow depth (**G**), Clark level (**H**), T stage (**I**), N stage (**J**), M stage (**K**) and clinical stage (**L**). (**M**) The C-index of ICDscore and clinical features of melanoma patients.
