# Supplementary material for: Machine learning-based construction of immunogenic cell death-related score for improving prognosis and response to immunotherapy in melanoma
Source: Aging (Albany NY). 2023 Apr 6;15(7):2667–88. doi: 10.18632/aging.204636 (PMC10120887; doi:10.18632/aging.204636)
Supplement: Supplementary Table 3 [file aging-15-204636-s004.pdf]

Supplementary Table 3. Correlation analyses between ICDscore and drug sensitivity.

Drug	R	P	Classification
JW-7-52-1	$R = 0.72$	$p < 2.2e{-16}$	undefined group
Roscovitine	$R = 0.71$	$p < 2.2e{-16}$	CDK inhibitor
Rapamycin	$R = 0.69$	$p < 2.2e{-16}$	mTOR inhibitor
CGP-60474	$R = 0.62$	$p < 2.2e{-16}$	CDK inhibitor
Erlotinib	$R = 0.62$	$p < 2.2e{-16}$	EGFR inhibitor
CAL-101	$R = 0.61$	$p < 2.2e{-16}$	PI3K inhibitor
STF-62247	$R = 0.59$	$p < 2.2e{-16}$	autophagy inducer
Sunitinib	$R = 0.59$	$p < 2.2e{-16}$	multi-kinase inhibitor
Z-LLNle-CHO	$R = 0.58$	$p < 2.2e{-16}$	Gamma secretase inhibitor
5-Fluorouracil	$R = 0.57$	p < 2.2e−16	chemotherapeutic agents
DMOG	$R = 0.57$	p < 2.2e−16	HIF-PH inhibitor
A-770041	R = 0.56	p < 2.2e−16	Lck inhibitor
AZD7762	$R = 0.56$	$p < 2.2e{-16}$	Chk inhibitor
THZ-2-49	$R = 0.56$	$p < 2.2e{-16}$	CDK inhibitor
Lestaurtinib (CEP-701)	$R = 0.55$	$p < 2.2e{-16}$	multi-kinase inhibitor
NU-7441	$R = 0.55$	$p < 2.2e{-16}$	DNA-PK inhibitor
PIK-93	$R = 0.55$	$p < 2.2e{-16}$	PI4K inhibitor
KIN001-260	$R = 0.54$	$p < 2.2e{-16}$	undefined group
UNC1215	$R = 0.54$	$p < 2.2e{-16}$	MBT inhibitor
BIX02189	$R = 0.53$	$p = 2.5e{-16}$	MEK inhibitor
Ruxolitinib	$R = 0.53$	$p < 2.2e{-16}$	JAK inhibitor
TPCA-1	$R = 0.53$	$p < 2.2e{-16}$	IKK-2 inhibitor
Y-39983	$R = 0.53$	$p < 2.2e{-16}$	ROCK inhibitor
Ponatinib (AP-24534)	$R = 0.52$	$p = 6.7e{-16}$	multi-kinase inhibitor
JW-7-24-1	$R = 0.52$	$p = 5e{-16}$	undefined group
KIN001-102	$R = 0.52$	$p = 3.8e{-16}$	Akt inhibitor
QL-XI-92	$R = 0.52$	$p = 1.1e{-15}$	undefined group
Tamoxifen	$R = 0.52$	$p = 1.1e{-15}$	Selective Estrogen Receptor Modulator
TG101348	$R = 0.52$	$p = 4.8e{-16}$	JAK inhibitor
Bexarotene	$R = 0.51$	$p = 5e{-15}$	RXR activator
SNX-2112	$R = 0.51$	$p = 1.7e{-15}$	HSP90 inhibitor
AICAR	$R = 0.5$	$p = 2e{-14}$	AMPK activator
Cyclopamine	$R = 0.5$	$p = 1.2e{-14}$	Hedgehog signaling inhibitor
VX-702	$R = 0.5$	$p = 8.1e{-15}$	MAPK inhibitor
