# Supplementary material for: Machine learning-based construction of immunogenic cell death-related score for improving prognosis and response to immunotherapy in melanoma
Source: Aging (Albany NY). 2023 Apr 6;15(7):2667–88. doi: 10.18632/aging.204636 (PMC10120887; doi:10.18632/aging.204636)
Supplement: Supplementary Table 5 [file aging-15-204636-s006.pdf]

Supplementary Table 5. Clinical information of patients from TCGA_SKCM dataset.

	TCGA-SKCM (n=448)	GSE65904 (n=214)	GSE22153 (n=57)	GSE54467 (n=79)
Age				
<60	238	80	21	44
>=60	210	130	36	35
Gender				
Female	168	89	26	29
Male	280	124	31	50
breslow depth (cm)				
<2	126		12	
>=2	222		26	
clark level				
I	5		3	
II	18		2	
III	75		11	
IV	162		15	
V	50		5	
T				
Tis-T0	30			
T1	41			
T2	76			
T3	89			
T4	145			
Tx	42			
N				
N0	222			
N1	73			
N2	49			
N3	55			
Nx	32			
M				
M0	402			
M1	22			
Stage				
0-II	225		0	58
III	169		3	20
IV	21		54	
Status				
Dead	220	102	47	48
Alive	227	108	7	31
